# 

**Published:** 1990-03

**Authors:** 


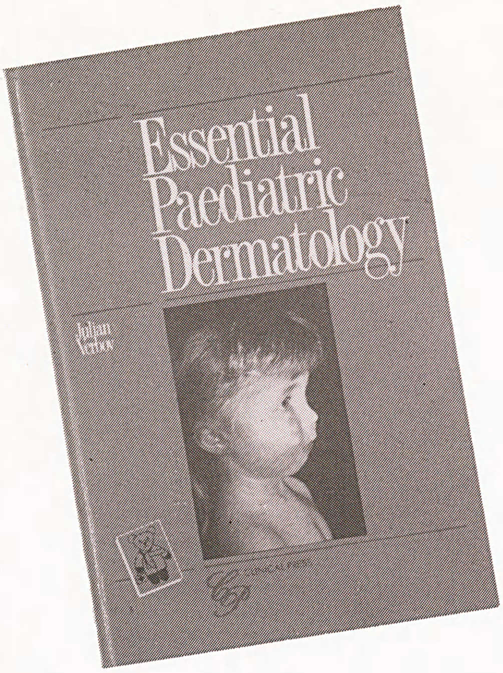

### Essential Paediatric Dermatology

*Julian Verbov, MD., FRCP., FIBiol.,*

Consultant Paediatric Dermatologist,
Royal Liverpool Children's Hospital

**Contents**
The approach to parent and child: The Newborn: Atopic and other dermatitis: Infections and infestations: Napkin area eruptions and erythematosquamous disorders: Hair and nails: Naevi and nodules: Connective-tissue disorders: Vascular disorders and drug eruptions: Genodermatoses: Non-hereditary bullous diseases and mastocytoses: Acne, trauma, light eruptions and pigmentation disorders: A selection of recommended prescribable proprietary preparations: Useful reading: Index

200 pages. Hardcover. 194 illustrations in full colour.

ISBN: 1 85456 000 5. Price £25.00

'This comprehensive and well-illustrated book aims to provide a wider understanding of skin diseases in children both in their diagnosis and management, and would be a useful addition to any practice library.'
*Pulse February 4th 1989.*

*'Full marks to Dr. Verbov, who has provided a crisp and lucid text suitable for trainees in medicine, paediatrics, and dermatology. In twelve chapters (starting with The Approach to the Child and Parent) most of the common skin conditions are described and beautifully illustrated in colour. Appendices deal with prescribing in dermatology and offer suggestions for further reading; there is a comprehensive index and each chapter concludes with selected contemporary references.'* **Lancet.**

---

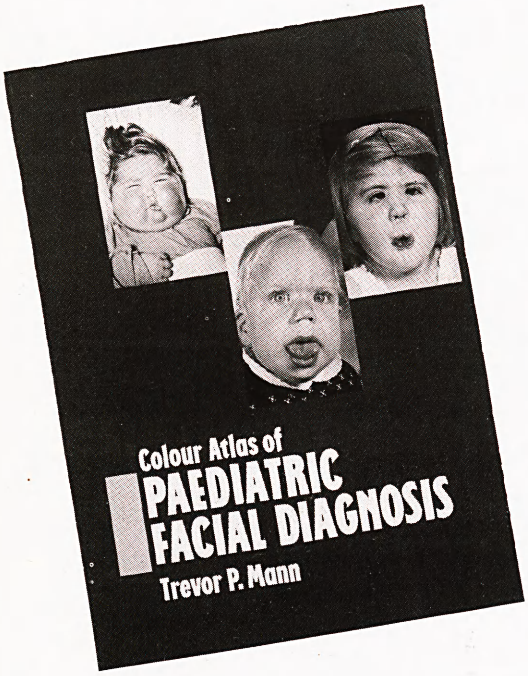

### Colour Atlas of Paediatric Facial Diagnosis

*Trevor P. Mann, MD., FRCP., DCH.,*

Honorary Consultant Physician,
Royal Alexandra Hospital for Sick Children,
Brighton

208 pages. Hardcover. 297mm × 210mm.

425 full colour illustrations.

ISBN: 1 85457 001 3. Price £70.00

This magnificent book demonstrates the methodology of facial diagnosis in children of all ages, including the new born. All common conditions in which facial diagnosis is important are to be found together with many diagnostic rarities. Each topic consists of a concise, informative and scholarly text, supported, where appropriate, by key references, many giving an historical perspective. There are over 400 high quality colour plates with descriptive legends, many of these analysing in some detail the individual features of a face regarded as abnormal or establishing a definitive diagnosis. The importance of recognising subtle expressive changes and 'facial signals' is considered in relation to emotional disorders. Throughout the book are tables listing important diagnostic clues, cranio-facial and otherwise.

*Clinical Press*

Please order from your usual bookseller or direct from Clinical Press Ltd., c/o Gazelle Book Services, Falcon House, Queen Square, Lancaster, LA1 1RN, UK

CP

**Editorial Committee**
Chairman of Editorial Board, Professor John Farndon
Editor—Mr M. G. Wilson
Assistant Editor—Dr Paul Goddard
Secretary—Mr Clive Ward
Members—Dr John Burton, Dr Kenneth Southgate

**Editorial Office**—*The Red House, 40 Coombe Lane, W.O.T., Bristol BS9 2BJ*
*Tel 0272 682320*

**Correspondents**
Dr Andrew Adam (Taunton)
Mr Martin Crosfil (Truro)
Dr Jack Davies (Bristol)
Professor Denis Pereira Gray (Exeter)
Dr Jose Jancar (Bristol)
Dr Michael Oliver (Barnstaple)
Professor Gordon Stirratt (Bristol)
Dr Michael Whitfield (Bristol)
Professor Gordon Wilcock (Bristol)

**Associated Societies**
Bristol Medico-Chirurgical Society
Clinical Society of Bath
Severn Faculty of the Royal College of General Practitioners
South West of England and Wales Society of Dermatology
South West Obstetricians and Gynaecologists Club
South West Orthopoedic Club
South West Paediatric Club
South West Radiologists Club
Surgical Club of South West England
Tamar Faculty of the Royal College of General Practitioners
Wessex Association of Clinical Pathologists
West Country Chest Society
West Country Physicians Club

**Published by**
Clinical Press
*15 Bucklands Drive,*
*Nailsea,*
*Bristol BS19 2PH*

**Typesetting by**
*Apek Typesetting*
*Avon House,*
*Nailsea,*
*Bristol BS19 2DJ*

**Printed by**
*Sutton Print, Paignton*

**THE WEST OF ENGLAND MEDICAL JOURNAL acknowledges with gratitude the help it receives from NUFFIELD HOSPITALS**

### WEST OF ENGLAND MEDICAL JOURNAL

*"What I know remains unknown, unless by me to others shown.* **Lucilius (180–102 BC)**

FORMERLY BRISTOL MEDICO-CHIRURGICAL JOURNAL

**NOTICE TO CONTRIBUTORS**
Contributions are invited especially from West of England authors on a variety of subjects, e.g. Original articles relating to the practice of medicine, reports on the contemporary medical scene, obituaries, historical accounts, recollections of colleagues and events worthy to be remembered and liable to be forgotten. An article of 2,000 words with 2 illustrations will occupy 2 pages and this is a suggested, though by no means an obligatory length. Articles are preferred in the form proposed by the International Committee of Medical Editors (Vancouver System)—pamphlet obtainable from Editor of BMJ., BMA. House, Tavistock Square, London, WC1H 9JR.

REGAINING BLADDER CONTROL

*Second edition*

Clinical Press

Eileen Montgomery, MCSP.,

For those suffering from incontinence on exertion or following pelvic surgery.

This important patient handbook contains a planned series of exercises aimed at restoring control of the bladder. The anatomical causes of incontinence are clearly explained and illustrated. The book also deals with correct breathing and a balanced diet which will not lead to constipation or to being overweight.

Surgeons, doctors and physiotherapists will be able to recommend this booklet to their patients.

28 pages. Soft cover. 11 line drawings. ISBN: 1 85457 003 X

Price inclusive postage and packing £2.50 per copy or £20.00 per pack of 10 copies.

Please order from your usual bookseller or direct from Clinical Press Ltd., c/o Gazelle Book Services, Falcon House, Queen Square, Lancaster, LA1 1RN, UK

Commonsense GERIATRICS

Keith Thompson, General Practitioner, London
Examiner for the Diploma in Geriatric Medicine

150 pages. Papercover. Fully illustrated. ISBN 1-85457-007-2. £12.50

Clinical Press

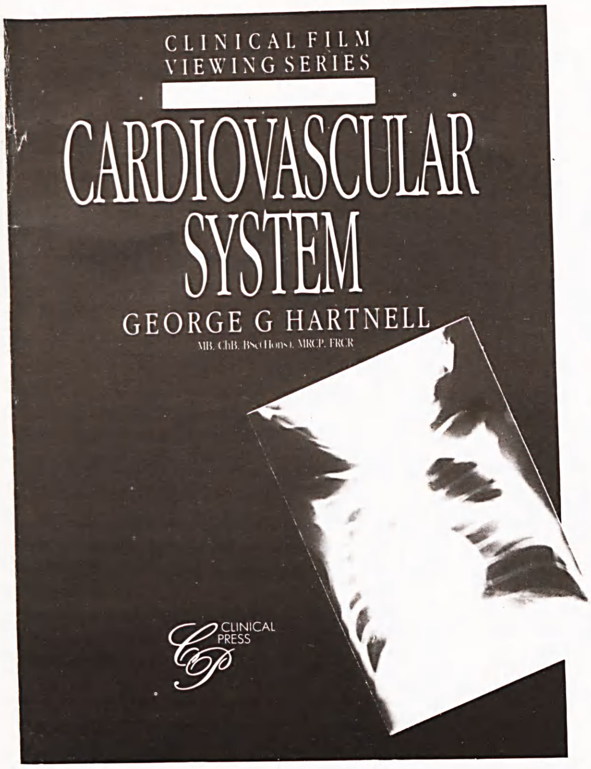

**Self assessment radiology**

### Clinical Film Viewing Series

Series Editor: Paul R. Goddard, MD., FRCR

Most postgraduate examinations now include radiographic interpretation. This may be presented as a slide show or as a film-viewing session. Radiographs and other imaging modalities are also used including those of the American Boards and professional societies and universities world wide.

In a typical examination situation, candidates are asked to view a film but are provided with minimal clinical information. They are then asked to give a written or oral report together with a sensible list of possible differential diagnoses.

This series of books on *Clinical Film Viewing* will assist candidates to prepare for this process. Each book examines a particular system or technique and promotes the reader's self-questioning about the film presented. The series provides a unique opportunity for self-assessment and learning in all the modern modalities of diagnostic imaging.

Each book consists of an introduction to the speciality or technique followed by eighty profusely illustrated cases typical of those presented in postgraduate examinations.

**To be published soon**

### CARDIOVASCULAR SYSTEM

George G. Hartnell, MB., ChB., BSc(Hons)., MRCP., FRCR.

'An invaluable learning book' from the Foreword by Professor E.R. Davies, *Department of Radiodiagnosis, Bristol Royal Infirmary.*

180 pages. 216mm x 138mm. Papercover. Over 100 illustrations. ISBN: 1-85457-0123-9. Price £15.00

### RESPIRATORY SYSTEM

Paul R. Goddard, BSc., MB BS, MD, DMRD, FRCR

200 pages 216mm x 138mm. Papercover. Over 110 illustrations. ISBN 1 85457 014 5

**Titles in the Series**

Moshy – General Information

Hartnell – Cardiovascular System

Goddard – Respiratory System

Kabala and Slack – Ultrasound

Lewis – Central Nervous System

Watt – Muskuloskeletal System

Virjee – Gasterointestinal System

Henson – Obstetrics and Gynaecology

Birch – Accident and Emergency

Banerjee – AIDS

Cook – Computed Tomography

Cook – Magnetic Resonance Imaging

---

**A concise guide for radiologists and radiographers.**

### LECTURE NOTES ON THE PHYSICS OF RADIOLOGY

Susan J. Armstrong, MB., ChB., MRCP., Registrar, Department of Radiodiagnosis, Bristol Royal Infirmary 'Susan Armstrong's book will be a companion throughout the course and a good read during the last anxious days before the exam. This is a book which will serve as a handy, concise and comprehensive reference in the years ahead. Written in an attractive style and free from superfluous verbiage, it will be immensely valuable to radiologists, radiographers, physicists, technicians and others professionally involved in radiology.' *From the Foreword by Professor Peter Wells, Honorary Professor, Bristol Royal Infirmary. Lecture Notes on the Physics of Radiology* is suitable for study for the higher qualifications in radiology and radiography.

CONTENTS: Atomic Structure and Radioactivity—The Production of X-Rays—X-Ray Generating Apparatus—Interactions between X-Rays and Matter—Dosimetry—Films and Screens—Image Quality—Fluoroscopy—Special Techniques—Computed Tomography—Ultrasound—Radionuclide Imaging—Magnetic Resonance Imaging—Quality Control—Radiation Protection—Bibliography—Index.

216 pages. Papercover. 216mm × 148mm. 98 illustrations. ISBN: 1-85457-010 2. £15.00.

*Clinical Press*

Please order from your usual bookseller or direct from Clinical Press Ltd., c/o Gazelle Book Services, Falcon House, Queen Square, Lancaster, LA1 1RN, UK

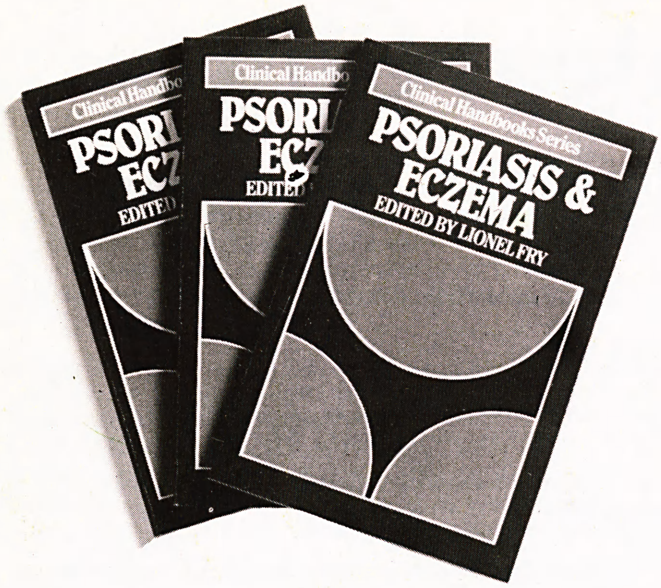

### Clinical Handbooks Series

This series is intended particularly for young doctors working in hospitals and primary care and for those training them. The series has been designed to cover growing points in medicine and the authors have been chosen, not just because of their expertise, but also because they are working both in hospitals and the community and are thus sensitive to the problems and needs of doctors in both areas.

*The first title in an important new series*

### PSORIASIS AND ECZEMA

*Edited by Dr. Lionel Fry, Consultant Dermatologist, St. Mary's Hospital, London.*

The book is intended to give some insight into recent advances in the pathogenicity of these diseases as well as their clinical features and management.

*Contributors:* Dr. B.S. Baker, Dr. L. Fry, Dr. C.E.M. Griffiths, Dr. A.V. Powles, Dr. R.J. Clayton, Dr. J.N. Leonard all at the Department of Dermatology, St. Mary's Hospital, London.

*Contents:* Psoriasis: Clinical Features (L. Fry) — The Treatment of Psoriasis (C.E.M. Griffiths) — The Aetiology and Pathogenesis of Psoriasis (B.S. Baker) — Eczema: Clinical Features (R.J. Clayton) — The Management of Eczema (J.N. Leonard) — The Aetiology of Eczema (A.V. Powles) — Index.

120 pages. Papercover. Fully illustrated in colour.

ISBN 1 85457 0002 1. £15.00

### Available soon

**HEART FAILURE** Edited by Gerald Sandler, Consultant Physician, Barnsley

Contents: Pathophysiology of Heart Failure. Treatment of Heart Failure. The Place of Surgery in the Management of Common Heart Conditions. The Clinical Aspects of Heart Failure.

100 pages approx. Papercover. ISBN 1 85457 008 0. £12.50 approx.

**DIABETES** Edited by Arun Baksi, Consultant Physician, St. Marys Hospital, Newport, IoW

Contents: What is diabetes mellitus – diagnosis and classification – Objectives of diabetes therapy – Medical treatment of non-insulin-dependent diabetes – Insulin treatment – The complications of diabetes – Index

100 pages. Papercover. Fully illustrated. ISBN 1 85457 005 6. £12.50

**SECOND OPINIONS IN INTERNAL MEDICINE** Edited by John Fry, General Practitioner, London, Gerald Sandler, Consultant Physician, Barnsley *and* Wesley Fabb, General Practitioner, Melbourne.

The problem of time wasting, unnecessary referral to specialist by primary care practitioners is universal. This unique book provides young doctors with a series of cases through which they can develop their judgement of whether to refer or not.

Contents: Jaundice – Resistant hypertension – Breathlessness – Dyspepsia – 'Mrs Neverwell' – Headaches – Swollen legs – Polyarthritis – Chest pain – 'Funny turns' – Dizziness – Backpain – Index

150 pages approx. Papercover. ISBN 1 85457 019 6. £12.50

**ORDER FORM**

Please order from your usual bookseller or direct from Clinical Press Ltd., c/o Gazelle Book Services, Falcon House, Queen Square, Lancaster, LA1 1RN, UK

Please send me

............. copy(s) of ..........................................................................

............. copy(s) of ..........................................................................

............. copy(s) of ..........................................................................

**Prepaid orders or credit-card orders will be supplied post free**

I enclose a cheque for ...................... (Payable to Gazelle Book Services)

OR

I authorise you to charge my Access/Barclaycard/American Express credit account

Card: ............................................ Number: ....................................

Expiry Date: .................................... Signature: ..................................

OR

Please invoice me at list price plus £1.50 per copy postage and packing

Name: (Please Print) ..........................................................................

Address: (Please Print) ........................................................................

